# Attitudes, knowledge and practices of healthcare workers regarding occupational exposure of pulmonary tuberculosis

**DOI:** 10.4102/phcfm.v6i1.597

**Published:** 2014-10-17

**Authors:** Lesley T. Bhebhe, Cornel Van Rooyen, Wilhelm J. Steinberg

**Affiliations:** 1 Department of Family Medicine, Faculty of Health Sciences, University of the Free State, South Africa; 2 Department of Biostatistics, Faculty of Health Sciences, University of the Frees State, South Africa

## Abstract

**Background:**

Healthcare-associated tuberculosis (TB) has become a major occupational hazard for healthcare workers (HCWs). HCWs are inevitably exposed to TB, due to frequent interaction with patients with undiagnosed and potentially contagious TB. Whenever there is a possibility of exposure, implementation of infection prevention and control (IPC) practices is critical.

**Objective:**

Following a high incidence of TB among HCWs at Maluti Adventist Hospital in Lesotho, a study was carried out to assess the knowledge, attitudes and practices of HCWs regarding healthcare-associated TB infection and infection controls.

**Methods:**

This was a cross-sectional study performed in June 2011; it involved HCWs at Maluti Adventist Hospital who were involved with patients and/or sputum. Stratified sampling of 140 HCWs was performed, of whom, 129 (92.0%) took part. A self-administered, semi-structured questionnaire was used.

**Results:**

Most respondents (89.2%) had appropriate knowledge of transmission, diagnosis and prevention of TB; however, only 22.0% of the respondents knew the appropriate method of sputum collection. All of the respondents (100.0%) were motivated and willing to implement IPC measures. A significant proportion of participants (36.4%) reported poor infection control practices, with the majority of inappropriate practices being the administrative infection controls (> 80.0%). Only 38.8% of the participants reported to be using the appropriate N-95 respirator.

**Conclusion:**

Poor infection control practices regarding occupational TB exposure were demonstrated, the worst being the first-line administrative infection controls. Critical knowledge gaps were identified; however, there was encouraging willingness by HCWs to adapt to recommended infection control measures. Healthcare workers are inevitably exposed to TB, due to frequent interaction with patients with undiagnosed and potentially contagious TB. Implementation of infection prevention and control practices is critical whenever there is a possibility of exposure.

## Abstrait

**Attitudes, Connaissances et Pratiques des membres du personnel soignant relatives à l'exposition du personnel soignant à la Tuberculose pulmonaire**.

**Historique:**

La Tuberculose nosocomiale (TB) est devenue un risque professionnel important pour le personnel soignant (HCWs). Les membres du HCW sont forcément exposés à la Tuberculose, en raison de l'interaction fréquente avec les patients ayant une tuberculose non diagnostiquée et potentiellement contagieuse. Il est essentiel de mettre en œuvre la prévention de l'infection et de faire des contrôles (IPC) quand il y a un risque d'exposition.

**Objectif:**

Suite à la haute incidence de Tuberculose parmi les HCW au Maluti Adventist Hospital du Lesotho, on a mené une enquête pour évaluer les connaissances, attitudes et pratiques des HCW en ce qui concerne la tuberculose nosocomiale et le contrôle des infections.

**Méthodes:**

C’était une étude transversale réalisée en juin 2011 à laquelle ont participé les HCW du Maluti Adventist Hospital, qui s'occupent des patients et/ou du frottis d'expectoration. On a fait l’échantillonnage stratifié de 140 HCW auquel 129 (92%) ont pris part. On a utilisé un questionnaire auto-administré et semi-structuré.

**Résultats:**

la majorité des répondants (89.2%) avaient une bonne connaissance de la transmission, du diagnostic et de la prévention de la Tuberculose, mais seuls 22% des répondants connaissaient la méthode appropriée de prélèvement des expectorations. Tous les répondants (100%) étaient motivés et désireux de mettre en œuvre les mesures de prévention et de contrôle des infections. Une proportion importante de participants (36.4%) a signalé de mauvaises pratiques de contrôle des infections où la majorité des pratiques inadéquates étaient les contrôles administratifs des infections (> 80%). Seuls 38.8% des participants ont signalé qu'ils utilisaient le respirateur approprié *N* = 95.

**Conclusion:**

Les mauvaises pratiques de contrôle des infections relatives aux risques professionnels d'exposition à la Tuberculose ont été démontrées, les pires étant les contrôles administratifs de première ligne des infections. On a identifié aussi des lacunes dans les connaissances. Cependant, il est encourageant de constater la volonté des HCW d'adopter les mesures recommandées de contrôle des infections. Les HCW sont forcément exposés à la Tuberculose, en raison de l'interaction fréquente avec les patients souffrant de Tuberculose non-diagnostiquée et potentiellement contagieuse. La mise en œuvre des pratiques de contrôle et de prévention des infections (IPC) est essentielle quand il y a la possibilité d'exposition à la contagion.

## Introduction

Tuberculosis (TB) has become a major, global public-health concern; in 2009,^1^ it claimed an estimated 1.7 million lives. In 2008, the Sub-Saharan Africa incidence rate of TB was 350 cases per 100 000 population, and 640 per 100 000 in Lesotho alone. Exacerbating factors are: the HIV epidemic, lack of proper facilities and resources for TB prevention, and lack of political will.^2, 3, 4, 5^ In the light of this high burden of TB, healthcare workers (HCWs) in high TB prevalence areas are inadvertently and inevitably exposed to TB, due to their constant interaction with patients with undiagnosed, untreated and potentially contagious TB in healthcare settings. In Kwazulu-Natal in South Africa, the incidence of TB among HCWs in public sector hospitals was higher than community-acquired TB.^6^

Despite TB being curable with the use of drugs, TB control depends on more than just chemotherapy. The ultimate prevention requires the integration of chemotherapy with infection prevention and control (IPC) measures to reduce transmission of TB in the general population and in health facilities.^7^ Knowledge and implementation of TB control measures by HCWs, and their compliance and willingness, remain the key factors in the effective management of healthcare-associated TB. Numerous comprehensive IPC guidelines have been designed for healthcare facilities, inclusive of low-resourced settings.^8, 9, 10^

In 2009, Maluti Adventist Rural District Hospital in Lesotho had seven HCWs diagnosed with healthcare-associated TB. That year, there were approximately 225 healthcare workers (inclusive of nursing students) working directly with patients and/or sputum. Thus, 3111 per 100 000 HCWs at Maluti Adventist hospital were newly infected with TB in that year. Those infected were of diverse profiles with one common factor: work-related exposure. Correct knowledge of the health problem, accompanied with the right attitude, can ultimately result in healthy practices and behaviour.^11^ Hence, the present study to evaluate knowledge, practices and attitudes of HCWs regarding healthcare-associated TB.

## Research methods and design

This was a cross-sectional descriptive study of knowledge, attitudes and practices of HCWs at Maluti Adventist Hospital in the Berea District of Lesotho, which is located 70 km south of the capital city, Maseru. These HCWs spent at least 2 h a day with patients in the ward or as outpatients, and were employed at the hospital for at least 6 months. All participants were 18 years and older, and gave written consent to participate in the study. The data were collected between June 01 and 30 2011. There were 337 workers at Maluti Adventist Hospital, of which, 225 were directly involved with patients and/or sputum at the time of the present study. A stratified sampling method was used to select 140 participants.

A self-administered questionnaire was used. This included: 13 demographic characteristics of the HCWs; 35 questions on knowledge (True/False or Yes/No type); 10 questions on attitudes (Yes/No type); and 12 questions on practices of HCWs regarding healthcare-associated TB infection (Yes/No type and two multiple choice-type questions). A pilot study was conducted on six participants, one from each of the six HCW population subgroups. No problems were identified and they were included in the study. The questionnaire was available in English and Sesotho, and distributed and collected by the researcher. The questionnaires were administered to each participant at different times, and participants were asked to be complete it without consulting a colleague. The study was approved by the Ethics Committee of the Faculty of Health Sciences of the University of the Free State (ECUFS NR 37/2011). Permission was granted by the Maluti Adventist Hospital Administration. Participation was on a voluntary basis and confidentiality was maintained throughout. Analysis of the data was performed by the Department of Biostatistics, University of the Free State. Total scores for each section for knowledge, attitude and practice were calculated and categorised as poor (< 39.0%), fair (40.0% – 69.0%) and good (70.0% +).

## Results

### Participant demographics

Of the 140 individuals selected for participation, 11 refused consent. This resulted in a final sample size of 129, which is a participation rate of 92.0% and corresponds to 57.3% of the HCW population. The distribution of the sample aligned well to the population strata of the HCWs at the hospital (see Table 1).

**TABLE 1:** Baseline characteristics of the sample (*n* = 129) of healthcare workers at Maluti Adventist Hospital.

Variable	Frequency (*n*)	%	Percentage in hospital
**Gender**		
Male	15	11.6	16.0
Female	114	88.4	84.0
**Profession type in hospital**		
General hand	7	5.4	4.0
Student nurse	70	54.3	55.1
Nurse assistant	29	22.5	20.9
Nurse attendant	9	7.0	10.2
Registered nurse	9	7.0	6.2
Medical doctor	5	3.9	3.6

Of the 129 respondents, 11.6% were male and 88.4% were female. The age of the respondents ranged from 19 to 60 years, whilst the mean and median age was 31 and 27 years, respectively. The duration of employment ranged from 1 to 37 years, with a mean duration of 6.7 years and a median of 3 years. Table 1 summarises the baseline characteristics of the study population and confirms the representativeness of the sample.

### Knowledge, attitudes and practices

In the knowledge section, 86.1% of the participants obtained a fair score (40.0% – 69.0%). Concerning attitudes, 93.0% of the respondents reflected good attitudes towards TB IPC measures, whilst 52.7% obtained a fair score for reported practices. Table 2 summarises the scores in the three respective areas.

**TABLE 2:** Levels of knowledge, attitudes and practices of Maluti Adventist Hospital healthcare workers towards tuberculosis infection control.

Variable	Frequency (*n*)	%
**Knowledge scores (***n*** = 129)**		
≤ 39%	14	10.9
40% – 69%	111	86.1
≥ 70%	4	3.1
**Attitudes scores (***n*** = 129)**		
≤ 39%	0	0.0
40% – 69%	9	7.0
≥ 70%	120	93.0
**Practice scores (***n*** = 129)**		
≤ 39%	47	36.4
40% – 69%	68	52.7
≥ 70%	14	10.9

TB, tuberculosis.

### Knowledge

Whilst the majority of respondents recognised pulmonary TB as being potentially contagious, 117 (90.7%) recognised airborne spread as the mode of transmission, whilst 12 (9.3%) gave other, inappropriate modes of transmission that included waterborne spread (1.6%) and direct contact (7.6%).

Most respondents were able to identify constitutional symptoms of TB; however, only 69 (53.5%) considered fever to be a symptom of TB. The majority of respondents recognised the link between HIV and TB, and knew that sputum examination was a major diagnostic tool; however, only 29 (22.0%) respondents were aware of the appropriate place for sputum collection. The majority of respondents were well acquainted with the circumstances around the treatment of PTB. The BCG (Bacille Calmette-Guérin) vaccination was identified by 75 (58.6%) respondents as not being a preventive measure against contracting pulmonary TB. Table 3 shows the participant responses on knowledge questions regarding transmission, predisposing factors, prevention, diagnosis and treatment of TB.

**TABLE 3:** Knowledge on tuberculosis transmission, predisposing factors, prevention, diagnosis and treatment by Maluti Adventist Hospital healthcare workers.

Variable (*n* = 129)	Appropriate knowledge		

	Symptoms	*n*	%
Airborne mode of transmission	-	117	90.7
Pulmonary TB as contagious type of TB	-	122	94.7
Pulmonary TB constitutional symptoms	Chronic cough	122	94.7
	Weight loss	102	79.1
	Fever	69	53.5
	Night sweats	109	84.5
HIV predisposition to contracting TB	-	123	96.1
Diagnosis of pulmonary TB by sputum smear	-	125	96.9
Appropriate place for collection of sputum	-	29	22.0
Six-month treatment duration	-	128	99.2
TB curable	-	126	97.7
Preventability of contracting pulmonary TB	-	117	91.4
BCG vaccination not preventive to contracting pulmonary TB	-	75	58.6

TB, tuberculosis; BCG, Bacille Calmette-Guérin; *n*, respondents.

### Attitudes

All respondents indicated that they were willing to change their habits and practices towards TB IPC methods, with 93.0% of them reporting positive attitudes. Table 4 contains the responses measuring HCW attitudes.

**TABLE 4:** Attitudes of healthcare workers towards preventive methods of nosocomial tuberculosis.

Variable (*n* = 129)	Yes		No		Not sure	
		
	*n*	%	*n*	%	*n*	%
Would you use a mask even though it is uncomfortable?	93	72.1	8	6.2	28	21.7
Would you be screened for TB if you had suggestive symptoms?	123	95.4	1	0.8	5	3.9
If diagnosed with TB, would you be willing to test for HIV?	113	87.6	7	5.4	9	7.0
If diagnosed with TB, would you be willing to complete treatment?	126	97.7	0	0.0	3	2.3
If you had a condition that predisposed you to contracting TB, would you be willing to change your work environment?	104	80.6	12	9.3	13	10.1
If malnourished, would you be willing to adjust your eating habits?	126	97.7	2	1.6	1	0.8
Would you be willing to provide adequate ventilation and sunlight, regardless of weather conditions?	116	89.9	1	0.8	12	9.3
Would you be willing to teach patients and co-workers about TB prevention?	124	96.1	0	0.0	5	3.9
Would you be willing to attend seminars for TB prevention?	122	94.6	2	1.6	5	3.9

TB, tuberculosis.

### Practices

Forty-seven (36.4%) of the respondents self-reported inappropriate practices (Table 2). The majority of the respondents (> 50%) reported poor administrative IPC control practice. Screening of TB patients for HIV was the exception, with 93.8% reporting appropriate practice. Environmental control measures, which are second-line IPC practices, had mixed responses with 35 (27.1%) respondents observing controls to avoid air recirculation into other (non-TB) wards. On personal protective measures, 92 (71.3%) respondents reported that they used masks, of which 54.4% used the N95 respirator (accounting for 38.8% of the entire sample size). Other personal protective measure practices were fair (Table 5).

**TABLE 5:** Administrative, environmental and personal tuberculosis infection control practices by Maluti Adventist healthcare workers.

Variable (*n* = 129)	Appropriate practice Knowledge	

	*n*	%
**Administrative control measures**		
Appropriate collection of sputum	23	17.8
Screening of suspicious TB patients in waiting areas (*n* = 127)†	25	19.7
Prioritising TB suspects for prompt service (*n* = 127)*	23	18.11
Screening TB patients for HIV	121	93.8
TB infection control education	34	26.4
**Environmental control measures**		
Isolation of patients, use of separate TB ward	101	78.3
Avoidance of air recirculation in non-TB wards	35	27.1
Open windows for ventilation and sunlight	89	69.0
**Personal protection measures**		
Personal protection by use of a respirator	92*	71.3
Use of appropriate respirator N-95 (*n* = 92)‡	50	54.4
Protective clothing usage	64	46.9
Hand hygiene	98	76.0
Disposition of linen infection control (*n* = 126)*	72	57.1

† 3 responses missing

‡ of the 92 respondents who use masks, 54.4% use the correct mask

* 2 responses missing

*n* respondents.

## Discussion

All HCWs managing inpatients and outpatients with a risk of healthcare-associated TB infection at this hospital were represented in the present study. The sample was comparable in composition to the HCW population strata of the hospital. A thorough understanding of the way in which TB is contracted remains critical for the implementation of IPC measures. The respondents of the present study confirmed sufficient knowledge about the transmission of PTB. The risk for TB transmission should be evaluated in all of the departments and areas where infectious TB patients might access care.

Eighty-nine percent of the respondents reported satisfactory knowledge of TB transmission, predisposing factors, prevention, diagnosis and treatment. The knowledge findings are similar to those of surveys conducted elsewhere in developing countries. Kiefer et al., whose scope of knowledge questions was similar to that of the present study, found that most participants scored between 57.8% and 85.0%, which falls within the fair and excellent range of knowledge scale of this study.^12^ Hoa et al.,^13^ with a similar profile of knowledge questions, found that rural Vietnamese health providers had a mean knowledge score of 67.0%. The mean knowledge score in the present study was 61.5%. In Iraq, Hashin et al.^14^ found the knowledge of TB among HCWs to be almost 100%. Most of the participants in their study had a diploma qualification as health professionals and the majority (73.0%) had been employed for more than 10 years. In contrast, the present study population included nursing students, and the median duration of employment was three years. In a Russian study by Woith et al.,^15^ the overall knowledge scores on TB and IPC practices were low for HCWs, which is in contrast to the present study.

Early diagnosis of infectious patients should result in a reduction of exposure of the HCWs in hospitals. If HCWs are able to identify the symptoms of TB, in the event that they are infected, they can seek help early and prevent morbidity and mortality associated with the infection. No studies can be cited of HIV prevalence among HCWs in Lesotho, but studies performed in South Africa have shown the prevalence of HIV among HCWs to be 15.7% and 11.5%.^16, 17^ The knowledge of HIV status and its association with TB is essential in order for susceptible HCWs to take relevant precautionary measures towards exposure reduction.

Knowledge gaps were identified in the present study, with only 22.0% of respondents knowing the appropriate sputum collection method. Critical knowledge gaps, despite good knowledge levels, were also found by Kiefer et al. in a study conducted in Peru.^12^ However, the actual knowledge gaps were different to the ones found in the present study. HCWs are frequently in contact with infectious patients and they facilitate the collection of sputum for both inpatients and outpatients. Sputum collection could thus predispose HCWs, as well as other patients, to healthcare-associated infection. Only 17.8% of the respondents reported collecting sputum correctly from patients, as recommended by Centres for Disease Control and Prevention (CDC) guidelines.^8^ This is likely a major risk for healthcare-associated infection.

TB control does not depend soley on chemotherapy, IPC measures and pathophysiology knowledge, but also on motivated HCWs with good attitudes towards IPC. Cooperation and team work is essential in the fight against TB, with HCWs who are willing to implement necessary changes and embrace new practices and recommendations. In the present study, 100% of the participants indicated that they were willing to implement preventive measures against healthcare-associated TB infection. A review study of several international studies support this finding of a high degree of willingness of HCWs to collaborate with TB national guidelines and infection controls.^18^ It needs to be borne in mind that these studies, as well as the present study, only tested the self-reported willingness of the HCWs to implement changes, which may differ from actual (eventual) practices.

The World Health Organization (WHO) proposed guidelines for IPC measures that are effective, affordable and can be implemented in resource-limited settings.^19, 10^ The CDC and WHO guidelines proposed the implementation of IPC measures at three levels: administrative, environmental and personal respiratory protection.^8, 10^ In order for the IPC methods to be effective, an IPC plan should be endorsed. In the IPC strategy, administrative controls are the cornerstone and first priority, whilst the other two are entirely dependent on administrative control for their effectiveness. Administrative controls reduce HCW and patient exposures to healthcare-associated TB infection. Poor IPC practices (as high as 36.4%) were measured in the present study, with poor administrative IPC measures constituting the majority. Administrative IPC measure implementation levels of less than 20.0% were reported. This is a huge pitfall, since these should be implemented at first contact with an infectious patient at a health facility. Delays in screening, diagnosis and treatment of TB due to failure of implementation of these controls increase the risk of healthcare-associated infection. Environmental IPC measures are dependent on infrastructural design, the use of ventilation and irradiation, which all require capital investment; however, simpler effective methods have been proposed based on adequate ventilation through the opening of windows.^10^ This is challenging, especially in Lesotho, where weather conditions are harsh, with winters that can record temperatures below 0 °C. This could possibly contribute to some HCWs not opening the windows for ventilation and keeping doors closed, explaining, to a degree, the low compliance reported with these IPC practices.

The findings regarding personal protective equipment were of concern. Biscotto et al. had similar findings, in which they found that HCWs infrequently used masks when performing procedures or attending patients with high risk of healthcare-associated infection. For example, of those attending TB isolation rooms, only 39.5% were observed to be using N95 respirators.^20^ This is comparable to the 38.8% in the present study who reported to be using respirators. On the other hand, unavailability of respiratory protective equipment could limit their use in the hospital, which was something not measured in the present study. Widespread and constant use of appropriate N95 respirators is regarded as impractical at Maluti Adventist Hospital due to the financial constrains resulting in failure to acquire them. In other studies, it has been noted that prohibitive costs in low-income countries in Africa result in failure of widespread use of N95 respirators for healthcare-associated TB prevention and control.^21, 22^ At Maluti Adventist Hospital, it was also noted that in some departments, the use of N95 respirators was limited to areas of perceived high risk for healthcare-associated TB infection only. This could have also contributed to a low percentage of the respondents reporting their usage in the present study.

### Limitations

The present study only measured variables that were self-reported by the HCWs and did not take into consideration the availability of resources (such as respirators) required for the implementation of IPC measures. This could have affected the results, as the respondents who reported not implementing these measures may have done so because of non-availability of resources. It is noteworthy to mention that the use of a self-administered questionnaire to measure practice is subject to response bias, as compared to the actual practices. Studies observing practices would yield more accurate data than using self-administered questionnaires.

## Conclusion

Despite the good general knowledge about TB that was shown by the HCWs, knowledge gaps regarding safe sputum collection were demonstrated. The HCWs reported to be willing to implement IPC practices. In this resource-limited setting, environmental controls and respiratory protective equipment implementation rates were reportedly low. Further studies should investigate why this is the case, and consider the availability of resources and other possible infrastructure constrains.

Administrative IPC measures should be the first-line pillar of infection control, and should receive more recognition in the studied setting. In the absence of appropriate IPC practices, healthcare-associated infections will inevitably remain a risk.
